# Influence of Control System Architecture on Mobile Robot Stability and Performance

**DOI:** 10.3390/s25237353

**Published:** 2025-12-03

**Authors:** Maciej Salwa, Izabela Krzysztofik

**Affiliations:** Faculty of Mechatronics and Mechanical Engineering, Kielce University of Technology, Aleja Tysiąclecia Państwa Polskiego 7, 25-314 Kielce, Poland; maciejsalwa@tu.kielce.pl

**Keywords:** control architecture, mobile robotics, PID control, microcontrollers

## Abstract

This paper presents an analysis of the impact of the control architecture in a mobile robot on the quality of regulation in real systems. Comparative studies were conducted for successive stages of the implementation of architectural improvements, such as optimization of RTOS resource utilization, the use of hardware mechanisms (DMP, DMA) for sensor data acquisition, and the optimization of subordinate controllers. The results showed that the final control quality depends not only on the controller tuning but also on the efficient management of the hardware and software resources of the control system. Retuning the PID controller after architectural modifications enabled the achievement of a better control quality index (IAE). The novelty of this work lies in demonstrating, through experimental evaluation, that embedded control architecture has a measurable and systematic impact on regulation quality in real systems. The obtained results indicate a significant relationship between control architecture and control performance, representing an important step toward bridging the gap between simulation studies and real-world implementations in mobile robotics.

## 1. Introduction

The control of self-balancing robots, particularly two-wheeled robots operating on the principle of an inverted pendulum, represents one of the classical problems in robotics and automation [1]. This topic has been the subject of numerous studies, covering aspects such as system modeling [2], control simulation [3], controller selection [4], optimization [5], and algorithm implementation and testing on real devices [6]. In many research works, the focus has been placed on the development and optimization of control algorithms under simulation conditions, followed by the transfer of the obtained results to a real object [7,8]. In many cases, there is a lack of proper analysis of the system’s behavior after transferring the algorithm from the simulation to the real device. Often, the process is limited merely to embedding the simulation results into a physical system, omitting practical implementation aspects such as the influence of the embedded system architecture on the robot’s performance and behavior.

An example can be found in the study [9], where a ready-made self-balancing robot based on the Arduino platform was used, and the PID control optimization was performed using LabVIEW software. Similar to many other studies on mobile robots, this research neglected the analysis of embedded system design aspects. In this context, the term embedded system design refers not only to hardware selection for control realization but also to implementation details such as interrupt management, processor thread handling, sensor data acquisition, and ensuring appropriate timing dependencies in the algorithm execution. In the case of a self-balancing robot, precise time management is crucial, especially when the system simultaneously executes multiple tasks (e.g., tilt angle measurement, motor control, and communication management).

On the other hand, articles addressing the influence of the physical control system [10] are usually of a general nature and do not show the impact of system architecture on the ability to reproduce simulation results. They tend to focus on hardware selection rather than on optimizing its utilization for specific tasks [11]. Similarly, works devoted to sensors in mobile robots usually discuss sensor capabilities without considering aspects such as the design of hardware and system interrupts to ensure efficient sensor usage [12]. The subject of selecting a microprocessor control architecture is occasionally discussed in textbooks [13] and scientific monographs [14], yet even there, studies exploring the impact of control architecture on control quality are missing.

This work aims to address that gap in the literature by emphasizing the impact of embedded system implementation on the robot’s performance in real-world environments. The goal is to demonstrate that the choice of hardware and the method of implementation significantly influence control quality and that different configurations can lead to noticeable differences in robot stability and responsiveness. Additionally, this paper presents an analysis of selected control algorithms in the context of their implementation on real devices such as microcontrollers, as well as an evaluation of how the embedded system architecture affects control quality under real operating conditions. As an example, a two-wheeled self-balancing robot equipped with inertial sensors for position estimation was used.

The structure of the paper is as follows: Section 2 presents the mathematical model of the robot. Section 3 describes the physical version of the system used, with particular attention to the sensory setup. Section 4 provides an analysis of the control architecture and the PID control system. Section 5 contains the experimental results and commentary on each architectural modification. Section 6 concludes the work with a summary of findings.

## 2. Mathematical Model of the Robot

A self-balancing robot is a commonly used implementation of the inverted pendulum concept. The inverted pendulum has the properties of a fourth-order model, which is multidimensional and can be treated as a typical control problem for analyzing modern control theories. Controlling such systems using classical methods is relatively challenging, mainly because it is a nonlinear system with two degrees of freedom (the robot’s angle and position) and only one control input. In the case of a two-wheeled robot, the part referred to as the pendulum is attached to the base, and the axis of rotation is the axis between the wheels, often called the torsion beam.

**Figure 1:**
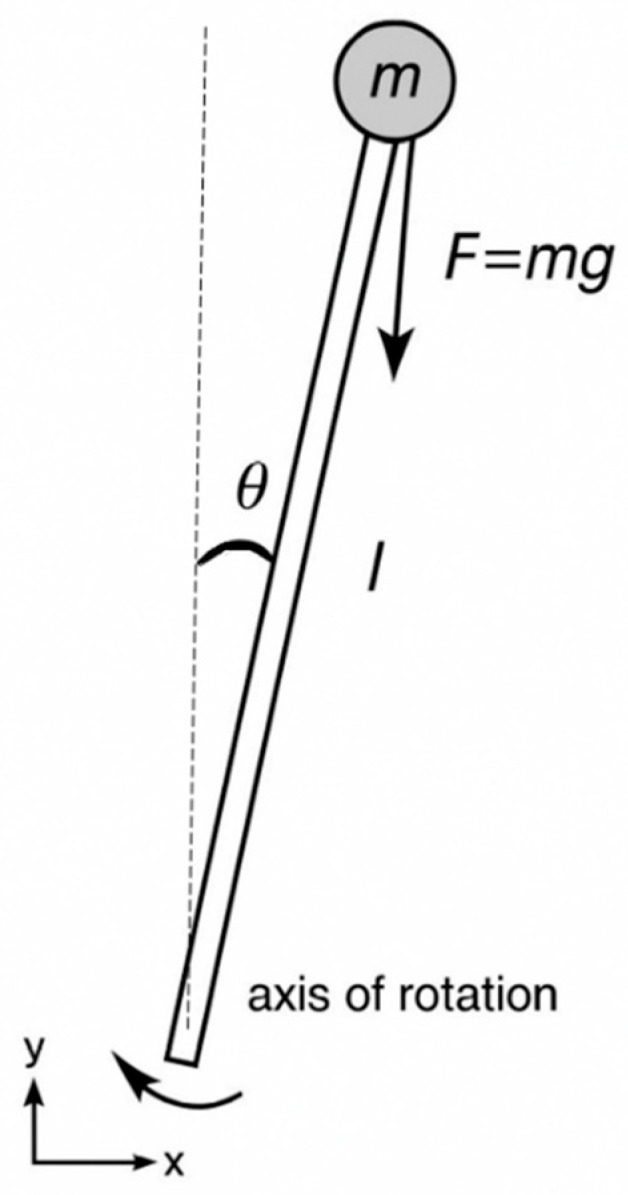
The concept of an inverted pendulum, where l—pendulum length, θ—pendulum deflection, m—pendulum mass, g—gravitational acceleration, and F—gravitational force.

Figure 1 shows a typical inverted pendulum—a system often considered theoretically, where it is generally assumed that the entire mass of the system is placed at the top of the pendulum.

**Figure 2:**
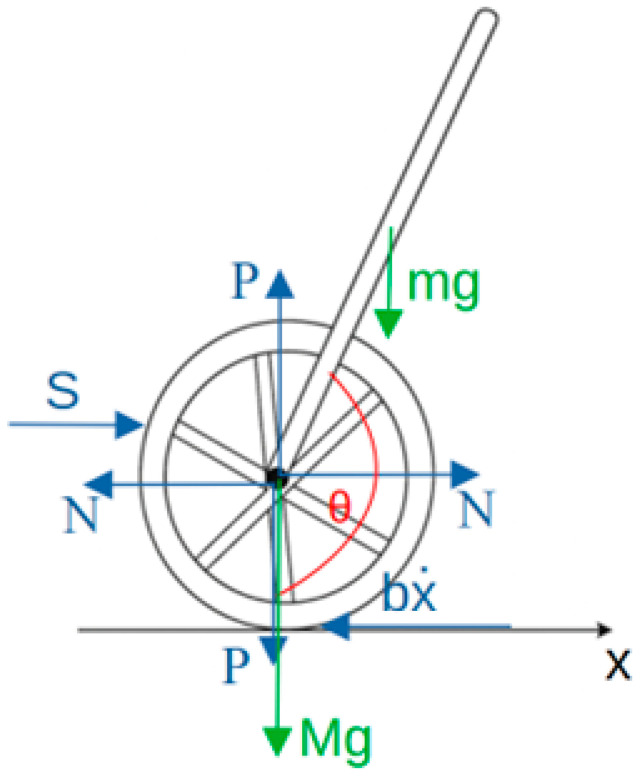
Force distribution for a two-wheeled self-balancing robot operating as an inverted pendulum.

Figure 2 schematically presents a two-wheeled self-balancing robot operating on the principle of an inverted pendulum, along with the distribution of forces acting on it.

At the joint point, additional forces resulting from the system’s interactions are introduced, where

M—mass of the wheels and the motor shafts attached to the wheels,

N—joint force acting in the horizontal direction,

P—joint force acting in the vertical direction,

S—resultant force acting along the X-axis, responsible for the robot’s displacement,

$\ddot{x}$—acceleration along the X-axis,

b—friction coefficient.

The sum of forces acting horizontally on the wheel–motor–shaft system based on Figure 2 can be expressed as the equation of motion:

(1) $$M\ddot{x}+b\dot{x}+N=S$$

Summing up the forces acting on the pendulum in the horizontal direction, we can write the equation for the reaction force in the joint as follows:

(2) $$N=m\ddot{x}+ml\ddot{\theta }cos\theta -ml\dot{\theta^{2}}sin\theta$$

Substituting Equation (2) into expression (1), we obtain one of the two equations of motion for the inverted pendulum realized by the self-balancing robot:

(3) $$\left(M+m\right)\ddot{x}+b\dot{x}+ml\ddot{\theta }cos\theta -ml\dot{\theta^{2}}sin\theta =S$$

To derive the second equation describing the considered control system of the inverted pendulum in the two-wheeled robot, we derive the equation for the sum of forces acting perpendicular to the ground at the joint. We obtain the following equation:

(4) $$Psin\theta +Ncos\theta -mgsin\theta =ml\ddot{\theta }+m\ddot{x}cos\theta$$

To eliminate the forces *P* and *N* from Equation (4), we sum the moments of forces with respect to the wheel axis (joint):

(5) $$-Plsin\theta -Nlcos\theta =I\ddot{\theta }$$

where

I—moment of inertia with respect to the wheel axis.

By solving the system of Equations (4) and (5), we obtain the second equation of motion for the analyzed control system of the inverted pendulum:

(6) $$\left(I+ml^{2}\right)\ddot{\theta }+mglsin\theta =-ml\ddot{x}cos\theta$$

## 3. Physical Object

**Figure 3:**
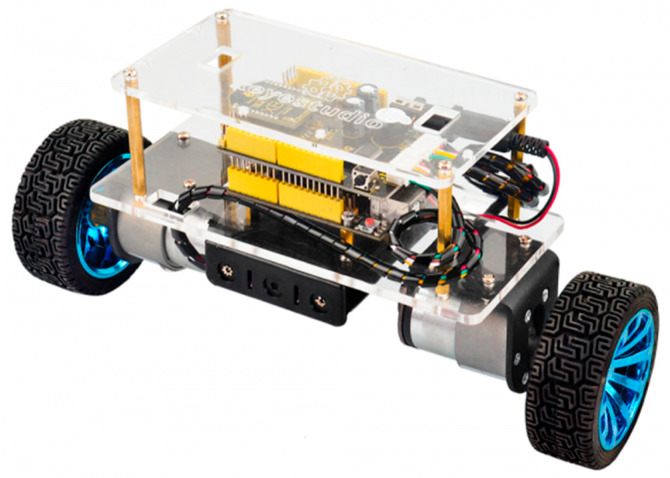
Keystudio self-balancing robot (Keystudio, Shenzhen, China) [15].

The research on the impact of control architecture on its quality was conducted using various self-balancing robot constructions, including a custom-built design. However, the experiments presented in Section 5 were carried out using a ready-made self-balancing robot, shown in Figure 3. The purpose of this approach was to increase the usability and reproducibility of the presented results, enabling other researchers to implement and further develop the proposed solutions more easily.

The self-balancing robot presented in Figure 3 is characterized by the following parameters declared by the manufacturer [15]:

1.  Motor parameters:
  - Operating voltage: DC 12 V.
  - Reduction ratio: 1:30.
  - No-load current: ≤100 mA.
  - No-load speed: 247 rpm.
  - Rated torque: 1.4 kg·cm (137.3 mN·m).
  - Rated speed: 160 rpm.
  - Rated current: ≤0.45 A.
  - Stalled torque: 5.5 kg·cm.
  - Stall current: 2.4 A.
  - Gearbox length: 22 mm.
2.  Operating voltage: DC 9–12 V.
3.  Motor driver chip: TB6612FNG.
4.  Posture detection: MPU-6050.
5.  Equipped with power control switch.
6.  Equipped with a Bluetooth control switch for serial communication.
7.  Total mass of the robot (with replaced controller): 1.5 kg.
8.  Wheelbase: 25.4 cm.
9.  Robot width (at the widest point): 7.5 cm.
10. Robot height: 11.5 cm.

In the robot, the control unit was replaced with an ESP32 module [16].

This module includes a microcontroller with significantly higher computational power, a built-in radio for wireless communication, and hardware counters enabling encoder data reading via DMA (Direct Memory Access)—thus offloading the main processor. It should be noted that the ESP32 is one of the most widely used microcontrollers today, and its price is comparable to that of the Arduino Uno platform originally used in the robot’s control system. The replacement was made to demonstrate how optimizing hardware capabilities can improve the overall control quality of the system.

From the control point of view, the most important sensors are as follows:

1. MPU-6050 [17]—an inertial MEMS sensor containing a three-axis accelerometer and a three-axis gyroscope, communicating via I^2^C. It supports both polling- and interrupt-based communication. The chip also includes an internal Digital Motion Processor (DMP) capable of performing calculations (e.g., filtering, calibration, orientation, and estimation) independently of the main microcontroller. According to the datasheet [17], the accelerometer noise level is about 400 µg/√Hz in a 10 Hz bandwidth. The measurement accuracy depends on the sensitivity settings (accelerometer/gyroscope range) and the calibration.
2. Hall-effect encoders integrated with the GM37-520 geared DC motors (1:30 reduction). The encoders are attached to both motors through the gearbox, which increases their effective resolution to 900 pulses per revolution. It should be noted that the gearbox introduces a minimal backlash—measured as less than 1°—which was neglected in the further analysis.

## 4. Control Architecture

### 4.1. Baseline Control Architecture

**Figure 4:**
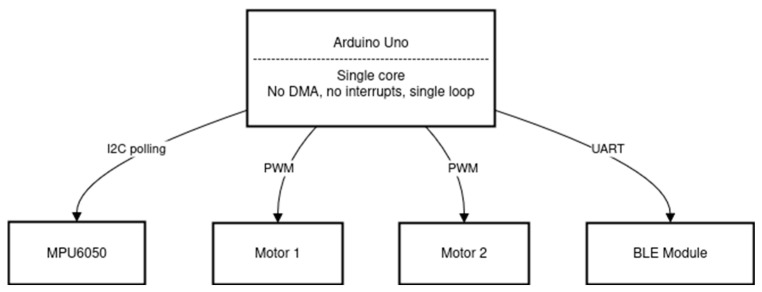
Mermaid diagram of the control architecture for Arduino Uno [own source].

The manufacturer of the robot provides control software designed for the Arduino Uno platform. The concept of this software is shown in Figure 4, using a Mermaid diagram—a standard notation for representing software system architectures.

The provided software uses a single control loop, which represents the simplest form of control, similar to that used in PLC controllers. During each iteration of the loop, the MPU6050 sensor is polled, and the Kalman filter implemented in software calculates the robot’s angular position. Next, PID regulation is performed and the resulting control signals (PWM signals) are sent to the motors. Subsequently, the robot’s position data is transmitted via the BLE (Bluetooth Low Energy) module. The process is then repeated—starting with a new MPU6050 sensor reading to begin the next iteration of the control algorithm.

**Figure 5:**
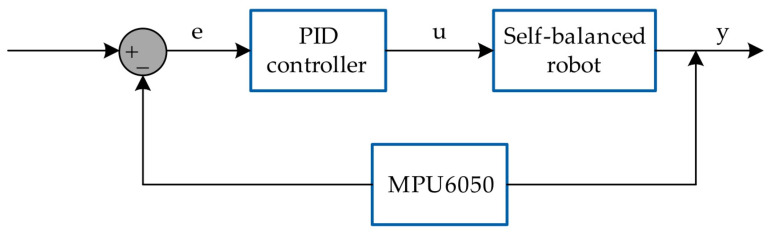
Control system of the self-balancing robot [own source].

As mentioned earlier, this is the simplest possible approach to implementing control using a microcontroller platform. The microcontroller used on the Arduino Uno platform is the ATmega328P [18], a chip based on the AVR architecture, characterized by limited memory, low computational power, and a lack of advanced peripherals such as DMA. A significant drawback resulting from these hardware limitations is the relatively long duration of the control loop, which affects the time step (dt). In simulation studies, dt is often a crucial factor in achieving precise control. However, in the manufacturer’s software, there is no synchronization mechanism for the main loop, meaning that dt is not constant—which leads to difficulties in tuning and optimizing the control system. The structure of the control system for the configuration described above is shown schematically in Figure 5.

The input to the control system is the angular position that the robot must maintain by moving its wheels. The feedback signal comes from the MPU6050 sensor readings.

### 4.2. Proposed Control Architecture

**Figure 6:**
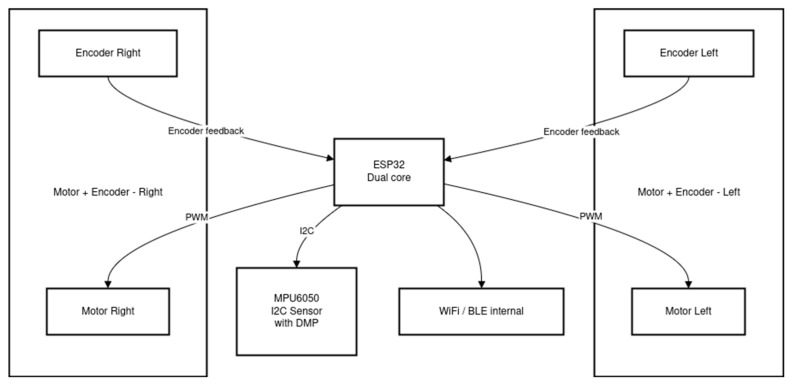
Mermaid diagram of the modified ESP32-based control architecture [own source].

The change in the control unit from Arduino Uno to ESP32 significantly increases the capabilities of the system compared to the original platform, offering two processing cores instead of one, greater computational resources, hardware counters integrated with DMA for encoder support, and built-in wireless communication. Figure 6 presents a Mermaid diagram of the modified control architecture.

The diagram itself does not show all the modifications introduced in the control architecture, which include the following:

1. The MPU6050 uses DMP (Digital Motion Processor) to perform filtering and compute the robot’s angular position, relieving the main processor of this task.
2. Both motors, represented as left and right control blocks, operate in independent control loops executed on the ESP32 at a higher frequency than the angular position control loop. Feedback from encoders is acquired through hardware counters utilizing DMA, further reducing CPU load.
3. Wireless communication is implemented via Wi-Fi, enabling real-time data transfer and robot tuning in Simulink/Matlab using MicroROS communication.
4. The entire system operates in real time using the ESP-IDF framework based on FreeRTOS. FreeRTOS is a real-time operating system that allows the active use of both processor cores: the first core handles the two control loops (the inner motor speed loop and the outer angular position loop), and the second core manages wireless communication.

This configuration results in a much smaller and more stable control loop time (dt) and allows data transmission via MicroROS, which is based on the DDS (Data Distribution Service) communication model. DDS is a standardized middleware protocol ensuring real-time data exchange. Its advantages include a peer-to-peer architecture (no central broker), providing high performance, scalability, reliability, and low latency through dynamic node discovery; a publish–subscribe communication model; data-centric design; and configurable Quality of Service (QoS) parameters, making it ideal for real-time autonomous systems such as robotic controllers. Real-time task management for the control algorithm is based on ESP32 hardware counters to ensure period repeatability. Communication tasks, performed on the second core, are managed by the FreeRTOS scheduler using system interrupts integrated with the IP stack handler implemented in esp-idf. The architectural changes introduce optimizations in sensor handling. Parameters such as data acquisition time and the architecture’s ability to respond to data in real time are optimized. This has an impact on the quality of input information to the system, which enables a better output state to be achieved in the physical robot.

### 4.3. Resource Allocation

The primary groups of real-time tasks are as follows:

- Outer attitude control angle loop (*θ*): high criticality for system stability; its execution period is tied to the update rate of the IMU readings,
- Inner wheel velocity control loop: requires rapid reaction to motor state changes and operates with a shorter period than the angle loop,
- Encoder processing tasks: responsible for accurate pulse counting; in the proposed design these are delegated to hardware counters with DMA, significantly reducing CPU load,
- Communication and logging tasks: necessary from a functional point of view but classified as soft real-time.

Priority assignment follows the rate-monotonic (RM) principle, where tasks with shorter periods receive higher priority. For preliminary schedulability evaluation, we use the classical Liu & Layland utilization bound for fixed-priority scheduling [19]. This is a popular approach which, despite the development of microprocessor technology over the years, is still used and remains practical [20]. For a set of n periodic, independent tasks with fixed priorities, the system is schedulable if its utilization satisfies the following:

(7) U′=n(2^(1/n)−1)

In the Liu & Layland schedulability test, the parameter *U* is defined as follows:

(8) $$U=\sum_{i=1}^{n}\frac{C_{i}}{T_{i}}$$

where

U′—the limit of processor utilization,

U—the total processor utilization required by the task set,

n—the number of periodic tasks,

$C_{i}$—the worst-case execution time (WCET) of task *i*,

$T_{i}$—the period of task *i*.

WCET values can be obtained by instrumenting the code and measuring the maximum observed execution time on the ESP32 platform. This provides a conservative estimate and allows verification of whether the control tasks can be executed without deadline misses. For architecture described in Section 4.2, the values are as follows:

- $C_{outer}$ = 0.25 ms (250 μs)—includes DMP read (I^2^C/SPI read latency, typically 50–150 μs depending on the bus + RTOS overhead), minimal post-processing (compensation and PWM preparation), and PID execution. An amount of 250 μs is a conservative starting point that provides a safe margin at *T* = 0.85 ms.
- $C_{inner}$ = 0.20 ms (200 μs)—assumes short PID for wheel speed, encoder read from DMA memory (i.e., minimal interrupts), and PWM setting. If the motors are controlled separately, it is worth considering the order of tasks (round-robin or priority assignment).
- $C_{encoder}$ = 0.02 ms (20 μs)—CPU cost is an occasional buffer read/sanity check; most tasks are performed by hardware DMA.

The calculated values for Equation (8) are as follows

(9) $$\begin{array}{c} \frac{C_{\mathrm{outer}}}{T_{\mathrm{outer}}}=\frac{0.25}{0.85}\approx 0.29411764705882353\\ \frac{C_{\mathrm{inner}}}{T_{\mathrm{inner}}}=\frac{0.20}{0.50}=0.4\\ \frac{C_{\mathrm{encoder}}}{T_{\mathrm{encoder}}}=\frac{0.02}{1.00}=0.02 \end{array}$$

The value of the U is equal to 0.71412. The calculated limit U′ from Equation (7) for n = 3 is as follows:

(10) $$U^{\prime }=\;3(21/3\;-\;1)\;\approx 0.77976$$

The condition for maintaining real-time operations is as follows:

(11) $$U\leq U^{\prime }$$

This is satisfied for the system under consideration, which is a single ESP32 core. The reserve is 0.06564.

Hardware offloading using the DMP and DMA directly affects the values of *Cᵢ*. Moving attitude estimation computations to the DMP reduces $C_{outer}$, while using DMA for encoder counting effectively eliminates CPU-side interrupt handling, lowering $C_{encoder}$. These reductions decrease total utilization U and reduce jitter and variance in the control loop periods, which directly improves closed-loop performance (lower overshoot, improved IAE detailed presented in Section 5).

With respect to multicore scheduling, assigning high criticality control tasks exclusively to one core and communication tasks to another reduces interference between hard and soft real-time activities. This isolation minimizes irregular delays caused by long-running communication operations and thus improves determinism.

## 5. Results

### 5.1. List of Experiments

To investigate the impact of control architecture design on system performance, a series of experiments was prepared in which architectural features described in Section 4.2 were added incrementally:

1. Optimization of PID controller parameters for angular position control using Simulink software and utilizing both cores of the microcontroller.
2. Use of DMP for reading the robot’s angular position.
3. Tuning of the inner control loop for the motor–encoder subsystem.
4. Comparison of control results and the system’s response to external disturbances.

### 5.2. Optimization of PID Controller Parameters for Angular Position Control Using Simulink

**Figure 7:**
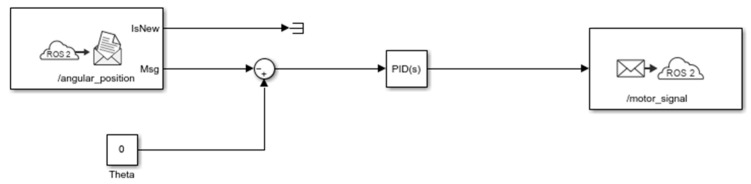
Control system implemented in Simulink.

This experiment focused on selecting optimal PID parameters for the robot’s angular position controller in order to achieve the best possible control performance using the baseline control architecture. In other words, for the baseline configuration shown in Figure 4, parameter optimization was carried out using search methods and dedicated Matlab/Simulink R2025b tools for PID tuning. A modification introduced at this stage was the replacement of Bluetooth communication with Wi-Fi, which enabled data transmission within an ROS2 network. To achieve this, the BLE module was replaced by a Wi-Fi module, and the microROS for Arduino library was used to exchange data with a PC running a Simulink-based data logging program. The PID controller itself was implemented in Simulink to facilitate tuning. This experiment served as a reference point for subsequent studies focused on improving robot control through architectural modifications. Figure 7 shows the control system implemented in Simulink for the data collected from the physical robot.

The input data were the cyclically computed angular positions of the robot obtained from the Kalman filter implemented in the robot’s controller. The output data from Simulink were motor control signals. The manufacturer’s default PID parameters for angular position control were Kp = 34, Ki = 0, and Kd = 0.62. The set control loop period was 5 ms. Based on experimental tuning, the optimized PID parameters are presented in Table 1.

**Figure 8:**
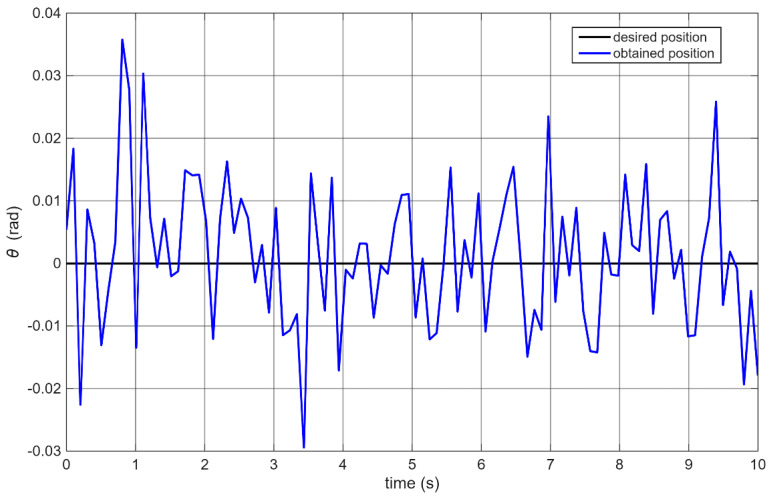
Control performance after PID parameter tuning.

It should be noted that the control loop period in the case of remote communication was extended depending on the needs of handling requests related to communication. Next, for the vertical position control task (where the desired angle *θ* = 0), an experiment was conducted in which only the calculated angular position was recorded. The PID algorithm was executed entirely by the robot’s controller. Figure 8 shows an example of the resulting control performance.

Since the experiments were conducted on real hardware, natural variation occurred between runs. To better evaluate the results, the IAE (Integral Absolute Error) criterion was selected, and each experiment was repeated 10 times, with the results averaged over 10 s intervals. The average IAE obtained was 9.43 [rad]. The standard deviation was 0.43 [rad]. The average maximum overshoot from 10 attempts was 0.042 [rad], with a standard deviation of 0.007. It should be noted that during this stage, the data sampling intervals were not perfectly regular due to the microcontroller being fully loaded and unable to maintain a constant control loop period. On average, the control loop took approximately 100 ms per iteration, with variations of ±20 ms. These data refer to the phase when PID tuning results were evaluated, and wireless communication involved only one-way transmission of angular position data.

### 5.3. Use of DMP for Reading the Robot’s Angular Position

In the next stage, the following architectural changes were introduced:

- Replacement of the controller with the ESP32 microcontroller (Espressif, Shanghai, China) described in Section 3.
- Integration of the FreeRTOS system (included by default in the ESP32 SDK).
- Utilization of the DMP filtering functionality built into the MPU6050 sensor (InvenSense Inc., Sunnyvale, CA, USA).

Introducing the ESP32 significantly increased computational power. FreeRTOS provided better task management and timing synchronization for the control loop. The built-in Wi-Fi allowed the same ROS2-based data logging as in the previous experiment. FreeRTOS was configured so that one core handled wireless communication, while the other was dedicated exclusively to control tasks and reading precomputed angular position data from the MPU6050 module.

As a result of these changes, the following improvements were achieved:

- More than fivefold increase in control loop execution frequency—period equal to 0.85 ms.
- Stabilization of the control loop period using hardware timers.
- Preservation of computational headroom on the microcontroller.
- Stable wireless communication independent of whether data were sent one-way or bidirectionally.

The addition of the ESP32 and the subsequent move to FreeRTOS brought a slight improvement in terms of stability and reduction in robot oscillations, which resulted from the incomplete utilization of the ability to react more quickly even to very small deviations from the vertical after they were calculated by the internal Kalman filter. The analyzed criteria for evaluating the regulation after the tests differed by less than 10% from the previous results. In order to achieve improvement in regulation, it was necessary to speed up the period of analyzing the position data. To this end, the use of DMP was introduced, thanks to which the data sent to the computing unit is in the form of ready-made information about the angular position. This allows for faster response and updates of control signals for the motors. It should also be noted that the filtering algorithm changed between experiments. In the previous test, the robot used a Kalman filter provided in the manufacturer’s software. In contrast, the MPU6050 DMP uses a complementary filter. The comparative evaluation of these filters was presented in [21]. While the Kalman filter generally offers higher accuracy, it also requires more computational resources. However, in the MPU6050 module, the complementary filter implemented within the DMP is factory-tuned for high accuracy. As demonstrated in [22], this built-in filter produced less noise than external filters (including the Kalman filter) and exhibited minimal deviation from reference values. Due to the use of the DMP and the other architectural improvements mentioned above, the IAE value (averaged over 10 trials) improved to 4.67 [rad]. The standard deviation was 0.14 [rad]. The average maximum overshoot from 10 attempts was 0.038 [rad], with a standard deviation for the max overshoot equal 0.005.

### 5.4. Tuning of the Inner Control Loop for the Motor–Encoder System

In the next stage, improvements were introduced to the encoder reading process. In the original code provided by the manufacturer, encoder pulses were read using hardware interrupts on the pins connected to the encoder’s signal lines. As a result, the microcontroller had to interrupt its control algorithm calculations to handle incoming encoder pulses. This is a highly error-prone approach in embedded system architectures, leading to several potential issues:

- Interrupts may disrupt the execution of the control algorithm.
- Simultaneous interrupts from both encoders may cause one to be missed.
- It complicates data flow management and makes software debugging more difficult.

Neglecting these issues can lead to false results that may accumulate and introduce errors into the control algorithm. Modern microcontrollers provide hardware mechanisms to improve encoder data collection, such as DMA-based pulse counting, which handles data acquisition independently of the main CPU. This enables a modular software structure, increasing flexibility for future code revisions—an important advantage from a software maintenance perspective. In terms of control quality, using DMA also allows for retuning the PID controller that regulates motor speed.

To optimize the motor PID parameters, an experiment lasting 15 s was prepared. The reference speed was changed in the following steps:

- from 0 to 120 rpm,
- at 5 s switched to −120 rpm,
- at 10 s switched to 240 rpm.

**Figure 9:**
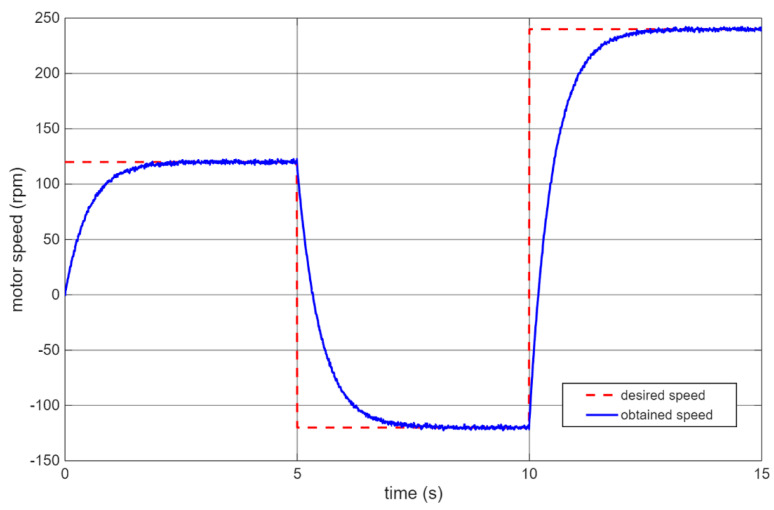
PID control of motor rotational speed in the self-balancing robot.

This scenario simulates typical operating conditions for a self-balancing robot, where rapid wheel reversals are required to maintain balance. The results of the motor speed control are presented in Figure 9.

The tuned PID parameters for motors are summarized in Table 2, showing significant differences from the manufacturer’s suggested values (Kp = 3.56, Ki = 0.072, Kd = 0).

After tuning the motor PID controller and optimizing the control architecture to the configuration shown in Figure 6, the main angular position controller (*θ* regulation) was retuned using the same Simulink-based approach described in Section 5.2. The newly obtained parameters are presented in Table 3.

**Figure 10:**
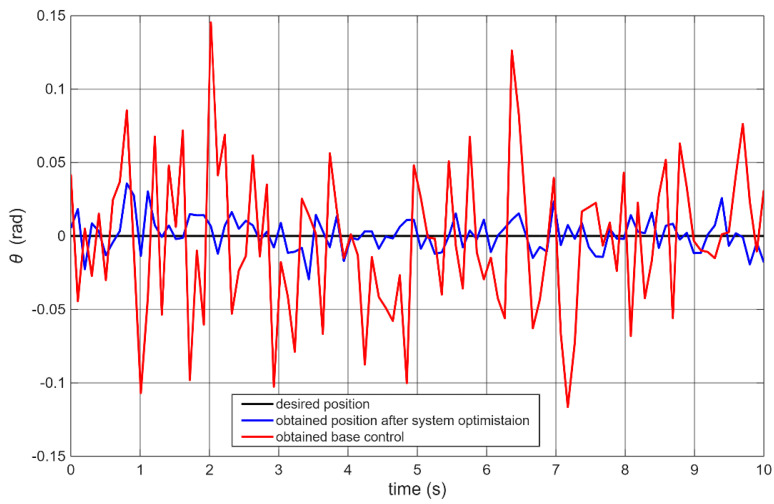
Comparison of self-balancing robot control performance for different software architectures.

These parameters differ from those initially presented in Table 1. The vertical position control experiment was repeated, resulting in an improved IAE value of 2.17 [rad] (average over 10 trials). The standard deviation was 0.13 [rad]. The average maximum overshoot from 10 attempts was 0.026 [rad], with a standard deviation for the max overshoot equal to 0.005. Figure 10 shows a graphical comparison of the robot stabilization performance for the baseline architecture (Figure 4) and the optimized architecture (Figure 6).

The blue curve represents the experiment conducted after tuning the motor PID controllers and retuning the main controller. To evaluate the impact of motor speed tuning alone, an additional test was performed using the baseline architecture but with the new motor PID parameters (Table 2). In this case, the robot became unstable and was unable to maintain an upright position.

### 5.5. Comparison of Control Performance–Response to External Disturbances

As a final experiment, control performance was compared between the baseline and the proposed architecture (Figure 6) under an external disturbance. The robot was struck by a pendulum-mounted ball to simulate impact or other disturbances typical in real-world operation. The pendulum ensured a consistent disturbance magnitude across trials.

**Figure 11:**
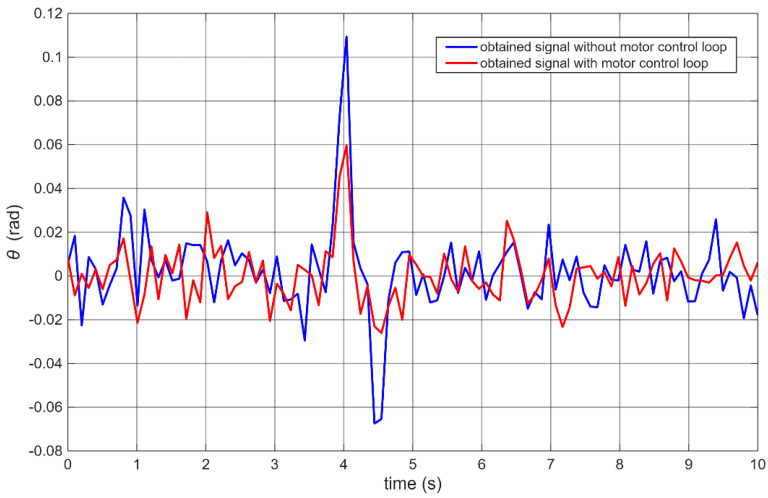
Comparison of robot response to external disturbance for different control architectures.

Figure 11 presents sample data of the robot’s angular position during collisions with the falling ball. For visual comparison, the time axis was scaled so that the impact peak occurred at the same moment in both cases.

In both tests, the ball was accelerated by gravity. The time axis was rescaled to align the moment of impact. For the robot with the modified architecture, a noticeably faster recovery from impact and nearly imperceptible negative overshoot (second peak) were observed—representing the corrective motion after the initial deflection.

## 6. Conclusions

This study investigated how successive architectural modifications in the embedded control system of a mobile robot affect regulation quality in real physical conditions. By systematically introducing improvements—such as optimized resource allocation through an RTOS, hardware offloading mechanisms including DMP and DMA, and adjustments to subordinate control loops—the experiments demonstrated that control performance depends not only on controller tuning but also significantly on the efficiency of the underlying computational architecture.

One of the key findings is that the parameters considered optimal for the baseline configuration may lose their optimality once architectural changes are introduced. Even small retuning adjustments, as shown for the angular PID controller (Table 3), resulted in measurable improvements in IAE, confirming that embedded system design and computational timing play an active role in shaping control performance.

The novelty of this work lies in experimentally demonstrating that architectural choices—often treated in the literature as implementation details—constitute an integral part of the control design process. The results provide evidence that consistent control performance cannot be achieved solely through simulation-based optimization; instead, attention must be paid to sensor utilization, timing determinism, and hardware resource management. In this sense, the presented methodology bridges the gap between simulation-orientated research and the practical realization of controllers on embedded platforms.

During the experiments, it was also observed that the most significant improvement in control quality resulted from optimizing sensor utilization, first through DMP-based pre-processing and later through DMA-based data acquisition. The influence of the hardware platform itself was less pronounced; however, switching to a more suitable MCU with dual cores enabled the effective use of these technologies and allowed their benefits to be fully exploited.

Naturally, the presented system also has limitations. The analysis focused on a specific microcontroller architecture and on classical PID-based regulation. More advanced controllers, such as MPC, LQR with state estimation, or adaptive control, were not considered, nor were alternative sensing modalities. Additionally, although the study highlights the role of timing and resource allocation, it does not explore automated methods for optimizing these aspects.

Future work may extend the presented methodology to a broader class of controllers and investigate automated architectural optimization strategies. Further studies may also include more sophisticated sensor fusion methods, real-time scheduling strategies, and multi-rate control structures. Such efforts could strengthen the generality of the presented conclusions and broaden their applicability to other embedded control systems.

## Figures and Tables

**Table 1:** Optimized PID parameters for angular position control.

Kp	Ki	Kd
36.45	0.42	1.13

**Table 2:** Optimized PID parameters for motor speed control.

Kp	Ki	Kd
23.76	0.52	2.15

**Table 3:** PID parameters for angular position control after motor tuning.

Kp	Ki	Kd
31.45	0.37	1.33

## Data Availability

The dataset used and analyzed in this study are available from the corresponding author upon reasonable request.
